# Cerebral hypoxia/ischemia selectively disrupts tight junctions complexes in stem cell-derived human brain microvascular endothelial cells

**DOI:** 10.1186/s12987-016-0042-1

**Published:** 2016-10-11

**Authors:** Shyanne Page, Alli Munsell, Abraham J. Al-Ahmad

**Affiliations:** Department of Pharmaceutical Sciences, School of Pharmacy, Texas Tech University Health Sciences Center, 1300 South Coulter Street, Amarillo, TX USA

## Abstract

**Background:**

Cerebral hypoxia/ischemia (H/I) is an important stress factor involved in the disruption of the blood–brain barrier (BBB) following stroke injury, yet the cellular and molecular mechanisms on how the human BBB responds to such injury remains unclear. In this study, we investigated the cellular response of the human BBB to chemical and environmental H/I in vitro.

**Methods:**

In this study, we used immortalized hCMEC/D3 and IMR90 stem-cell derived human brain microvascular endothelial cell lines (IMR90-derived BMECs). Hypoxic stress was achieved by exposure to cobalt chloride (CoCl_2_) or by exposure to 1 % hypoxia and oxygen/glucose deprivation (OGD) was used to model ischemic injury. We assessed barrier function using both transendothelial electrical resistance (TEER) and sodium fluorescein permeability. Changes in cell junction integrity were assessed by immunocytochemistry and cell viability was assessed by trypan-blue exclusion and by MTS assays. Statistical analysis was performed using one-way analysis of variance (ANOVA).

**Results:**

CoCl_2_ selectively disrupted the barrier function in IMR90-derived BMECs but not in hCMEC/D3 monolayers and cytotoxic effects did not drive such disruption. In addition, hypoxia/OGD stress significantly disrupted the barrier function by selectively disrupting tight junctions (TJs) complexes. In addition, we noted an uncoupling between cell metabolic activity and barrier integrity.

**Conclusions:**

In this study, we demonstrated the ability of IMR90-derived BMECs to respond to hypoxic/ischemic injury triggered by both chemical and environmental stress by showing a disruption of the barrier function. Such disruption was selectively targeting TJ complexes and was not driven by cellular apoptosis. In conclusion, this study suggests the suitability of stem cell-derived human BMECs monolayers as a model of cerebral hypoxia/ischemia in vitro.

**Electronic supplementary material:**

The online version of this article (doi:10.1186/s12987-016-0042-1) contains supplementary material, which is available to authorized users.

## Background

The blood–brain barrier (BBB), a component of the neurovascular unit, constitutes a crucial biological barrier in the maintenance of brain homeostasis by restricting the diffusion of solutes and toxins to brain parenchyma. The presence of such barrier is supported by brain microvascular endothelial cells (BMECs), which are present in the cerebral microvasculature. BMECs provide both a physical (tight junctions complexes) and a chemical barrier (drug and nutrient transporters), which tightly regulate the diffusion of small molecules between the blood and brain. However, the integrity of the BBB is compromised in several neurological diseases including multiple sclerosis [1], neurodegenerative diseases [2–5], and stroke [1, 6, 7].

Stroke constitutes the fifth leading cause of death in industrialized countries and is a leading cause of disability [8]. The majority of stroke events are classified as an ischemic, marked by an abrupt decreased perfusion in a defined brain region, resulting in an impairment of both oxygen and nutrient supply. This ultimately leads to the onset of a cerebral hypoxic/ischemic (H/I) injury.

BMECs are the first cell type of the neurovascular to sense hypoxia and respond to such injury by disrupting barrier function. Such disruption will eventually lead to a vascular leakage. The mechanisms by which H/I impacts barrier function have been extensively studied in rodents and non-human primates [9–17], yet the literature showing similar outcomes at the human BBB remains unclear.

In this study, we investigated the effect of H/I on a stem cell-derived model of the human BBB using the IMR90-c4 induced pluripotent stem cell line [18–21] and compared their response to hCMEC/D3, an immortalized human BMEC line commonly used in the literature [22].

## Methods

### Cell culture

hCMEC/D3 cell line [22] was purchased from Millipore (EMD Millipore, Billerica, MA, USA) and maintained following established manufacturer protocol. IMR90-c4 induced pluripotent stem cell (iPSC) cell line [18] was purchased from WiCell (WiCell, Madison, WI). IMR90-c4 iPS cell line was maintained in mTeSR1 (Stem Cell Technologies, Vancouver, BC, USA) and grown on hPSC-qualified Matrigel (Corning Inc., Corning, NY, USA). The IMR90 cell line was differentiated into BMECs (iPSC-BMECs) following the differentiation protocol established by Lippmann and colleagues [20, 21] and summarized in Additional file 1: Figure S1. In brief, cells were seeded at 20,000 cells/cm^2^ 5 days before differentiation and maintained in mTeSR medium. Five days after seeding, BMECs differentiation was set using unconditioned maturation medium (UMM) following the same composition as previously described: DMEM/F12 with 15 mM HEPES supplemented with 20 % KO serum replacement, 1 % MEM non-essential aminoacids, and 0.5 % Glutamax I, (ThermoFisher, Waltham, MA, USA) and 0.1 mM β-mercaptoethanol (Sigma-Aldrich, St Louis, MO, USA) for 6 days. After such differentiation, IMR90-derived BMECs were incubated in presence EC differentiation medium (EC serum free medium (ThermoFisher), supplemented with 1 % platelet-poor derived plasma serum (ThermoFisher), 20 µg/mL human basic fibroblast growth factor (R&D Systems) and 10 µM all-trans retinoic acid (Sigma-Aldrich) for 2 days. After 8 days of differentiation, cells were enzymatically dissociated and seeded at a density of 10^6^ cells/cm^2^ on 12-well Transwell polyester cell culture inserts (0.4 µm pore size) coated with collagen from human placenta (Sigma-Aldrich) and fibronectin from bovine plasma (Sigma-Aldrich) at concentrations of 80 and 20 µg/cm^2^. After 24 h (day 9), BMECs were maintained in EC differentiation medium containing only 1 % platelet-poor plasma-derived serum. All experiments were carried out 48 h after seeding.

**Fig. 1:**
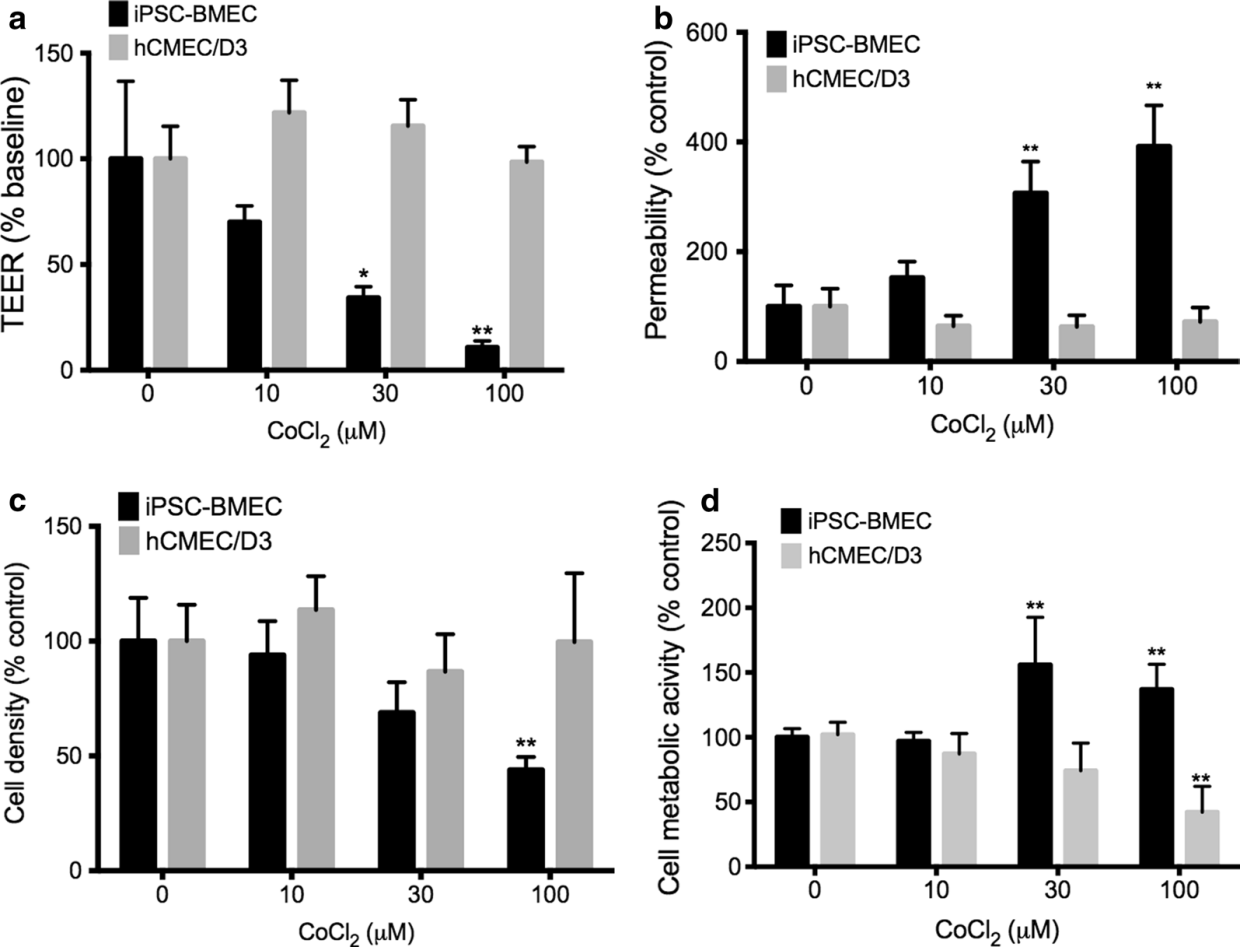
Cobalt chloride induces barrier disruption in iPSC-derived BMECs but not in hCMEC/D3 monolayers. Cells were treated with cobalt chloride (CoCl_2_) for 24 h and were compared to untreated cell monolayers. Changes in the monolayer integrity were assessed by TEER (**a**) and by sodium fluorescein (**b**). Note the decrease in barrier function in IMR90-derived BMECs (iPSC-BMEC) versus the hCMEC/D3 monolayers. **c** Cobalt chloride induced decrease in adherent cell density in iPSC-BMEC at high concentrations (100 µM). * and ** denotes *P* < 0.05 and P < 0.01 versus control (untreated) groups. **d** Metabolic activity following treatment with CoCl_2_ did not induce cell toxicity in IMR90-derived BMECs, as we noted no decrease versus control group. Mean ± SD, n = 3 for each group, * and ** denotes *P* < 0.05 and P < 0.01 versus control (untreated) groups

### Barrier function

Cells were grown on 12-well Transwell polyester membranes (Corning Inc.) coated with 30 µg/cm^2^ collagen I (Sigma-Aldrich, St Louis, MO, USA) or with 80 µg/cm^2^ collagen supplemented with 20 µg/cm^2^ fibronectin (Sigma-Aldrich) mixture to allow hCMEC/D3 and IMR90-derived BMECs to attach respectively. Monolayer tightness was assessed by measuring transendothelial electrical resistance (TEER) using an EVOM STX2 chopstick electrode (World Precision Instruments, Sarasota, FL, USA). Paracellular permeability was assessed in monolayers by measuring the diffusion profile of 1 µM of sodium fluorescein (Sigma-Aldrich) using the clearance method described by Perriere and colleagues [23]. Fluorescence was assessed using a Synergy MX2 ELISA plate reader (Bio-Tek Instruments, Burlington, VT, USA). Average TEER and permeability values of both monolayers can be found in Table 1. TEER and permeability values from untreated monolayers were used to normalize experimental values expressed as % of control. Untreated group experimental values were arbitrarily set to 100 % control.

**Table 1:** Biological values of hCMEC/D3 and IMR90-derived BMECs under resting conditions

Cell type	hCMEC/D3	IMR90-derived BMECs
Cell density (× 10^3^ cells/cm^2^)	128 ± 21	98 ± 19
TEER (Ω cm^2^)	40 ± 14	392 ± 113
Pe fluorescein (10^−4^ cm/min)	19 ± 8	2.44 ± 1.12
Metabolic activity (OD 490 nm)	1.26 ± 0.45	0.89 ± 0.34

Average cell density, TEER, fluorescein permeability (Pe) and metabolic activity (MTS absorbance at 490 nm wavelength). Mean ± SD, n = 18 for TEER and Pe values

### Cobalt chloride and hypoxic treatment

Cobalt chloride (CoCl_2_, MP Biomedicals, Santa Ana, CA, USA) was freshly dissolved in complete cell medium at a stock concentration of 100 mM and further dissolved to achieve the concentrations of 10, 30 and 100 μM. Cells were incubated in presence of CoCl_2_ for 24 h. For the hypoxic experiments, cell medium was replaced with fresh medium and cells were incubated in a normobaric hypoxic C-chamber (Biospherix, Laconia, NY, USA) set at 1 % O_2_, 5 % CO_2_ and maintained at 37 °C for 6 or 24 h. In experiments involving oxygen deprivation (OD), cell medium was replaced with Dulbecco’s Modified Eagle Medium (DMEM) containing 1 g/L d-glucose and 1 % platelet-poor derived serum (Alfa Aesar, Ward Hill, MA, USA). In GD and OGD experiments, glucose-free and pyruvate-free DMEM were used instead of DMEM containing 1 g/L d-glucose.

### Immunocytochemistry

Cells were grown to confluence on 48-well coated plates and quickly washed with ice-cold phosphate buffered saline (PBS) solution and fixed with either 4 % paraformaldehyde (Electron Microscopy Sciences, Hatfield, PA, USA) or cold methanol (ThermoFisher) and processed as previously described [20, 21].

### Cell density and viability assay

Cell density was assessed on monolayers by Trypan-blue exclusion based assay. In brief, monolayers were briefly washed with ice-cold PBS and incubated in presence of accutase (corning) or 0.25 % trypsin–EDTA (ThermoFisher) for 5 min followed by a centrifugation at 1000 rpms for 5 min. Cells were resuspended in 0.5 mL PBS and counted using a Countess II automated cell counter (ThermoFisher). Cell viability was assessed by MTS-based CellTiter 96^®^ Aqueous (Promega, Madison, WI, USA). After treatment, cells grown on 96-well plates were incubated in presence of MTS reagent for 1 h. Absorbance was measured at 490 nm and corrected against unconditioned medium containing the same amount of MTS. Absorbance values from all samples were normalized against their respective control samples.

### Statistics

All experiments were performed using cells coming from at least three independent experiments (distinct cell passages, each experiment was performed with two technical replicates). Statistical analysis was performed using one-way ANOVA followed by a post hoc analysis using Dunnett’s test with the control group as the reference group. P values lesser or equal to 0.05 were considered statistically significant.

## Results

### CoCl_2_ induces barrier disruption in IMR90-derived BMECs but not in hCMEC/D3 monolayers

In order to demonstrate the suitability of IMR90-derived BMECs (iPSC-BMECs) as an in vitro model of cerebral hypoxia/ischemia, it is important to show that such cells can respond to known hypoxic stimuli. Thus, we first assessed the cellular response of iPSC-BMEC monolayers to chemical hypoxia (Fig. 1) by exposing them to different concentrations of cobalt chloride (CoCl_2_), a common hypoxia-mimetic chemical used in various cell types [9, 24–29]. We compared iPSC-BMECs response to hCMEC/D3 cell monolayers, a human brain microvascular endothelial cell line commonly used in the literature [22, 30–32]. It should be noted that these cell monolayers have lower baseline TEERs and higher sodium fluorescein permeabilities that the iPSC-BMEC monolayers (Table 1).

Upon exposure of 24 h to CoCl_2_, there was a decrease in the iPSC-BMECs barrier function as noted by a significant decrease in TEER (Fig. 1a) in cells treated with CoCl_2_ at 30 and 100 µM. However, hCMEC/D3 failed to display any changes in barrier function even when exposed at 100 µM (a dose known to trigger a barrier breakdown in rat brain endothelial cell monolayers [9]). To further confirm this observation, we investigated changes in sodium fluorescein permeability in both iPSC-BMECs monolayers and hCMEC/D3 following CoCl_2_ treatment (Fig. 1b). As observed in our previous experiment, CoCl_2_ treatment affected the barrier integrity in iPSC-BMECs at 30 and 100 µM, with a 3- to 4-fold increase in paracellular permeability to sodium fluorescein compared to untreated cells.

### CoCl_2_ treatment differentially affects cell density and metabolism in hCMEC/D3 and IMR90-derived BMEC monolayers

Because CoCl_2_ treatment can induce cell apoptosis in various mammalian cell lines [33–36], we investigated changes in cell viability following CoCl_2_ treatment using MTS and Trypan-blue exclusion assays (Fig. 1c, d). After 24 h of CoCl_2_ treatment, we did not note a significant decrease in hCMEC/D3 cell density (Fig. 1c). However, we noted a 50 % decrease in cell density only in iPSC-BMEC group exposed to 100 µM, suggesting a detrimental effect of CoCl_2_ on the monolayer integrity. However, no change in hCMEC/D3 cell monolayer density was observed, even at the highest CoCl_2_ concentration.

Attempts to use trypan blue exclusion assay only provided information on adherent cells remaining after CoCl_2_ treatment. Furthermore, the additional mechanical stress applied to assess cell viability in non-adherent cells using such method may also interfere with the method accuracy. Therefore, we consider such method not suited for cytotoxic assays. Notably, tetrazolium-based cytotoxic assays directly assess changes in mitochondrial activity and can be directly applied to adherent and non-adherent cells. Therefore, we investigated changes in cell viability following CoCl_2_ treatment using an MTS-based assay (Fig. 1d), an assay commonly used to assess cytotoxicity by measuring changes in cell metabolic activity [37]. Notably, we did not notice changes in cell viability in iPSC-BMEC monolayers following exposure to CoCl_2_ compared to control. We even noted an increase in cell viability following 30 µM and 100 µM treatment. In contrast, we noted a dose-dependent decrease in cell viability in hCMEC/D3 monolayer with a 50 % decrease in cell metabolism.

**Fig. 2:**
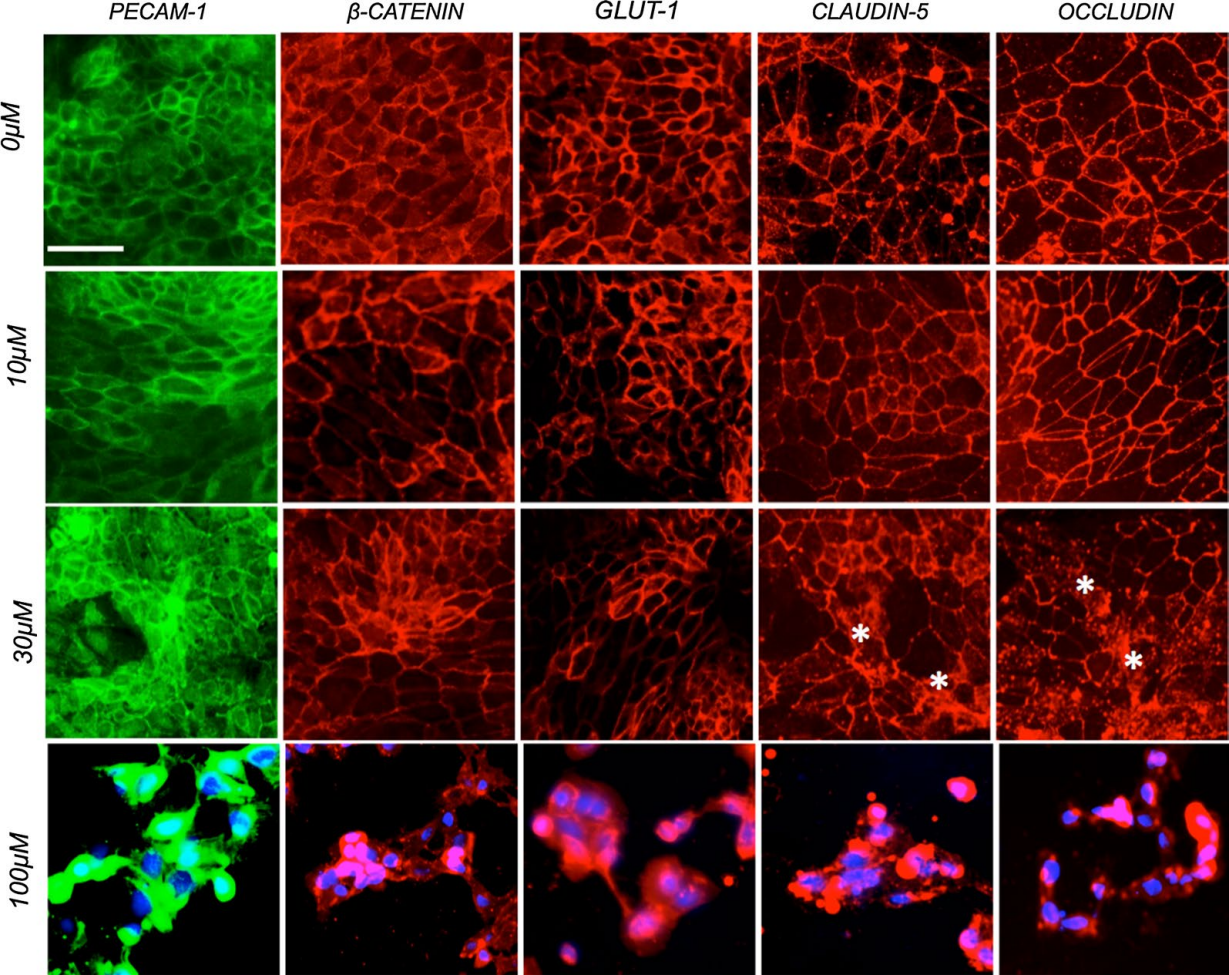
Cobalt-chloride treatment impact monolayer integrity in iPSC-derived BMECs. Representative immunocytochemistry micrograph pictures of IMR90-derived BMECs monolayers in presence of CoCl_2_. Cells were treated with different CoCl_2_ concentrations. Note the presence of irregular patterning in TJ proteins (claudin-5 and occludin) as marked by asterisks at 30 µM treatment and a complete disruption at 100 µM. *Scale bar* = 20 µm

To further understand how CoCl_2_ impacted barrier function in iPSC-BMECs, we investigated changes in cell junction integrity by immunocytochemistry (Fig. 2). At 30 µM, we observed alterations in the distribution of tight junction complexes (claudin-5 and occludin) with a loss of immunostaining at the cell borders and a relocalization into cell cytoplasm. Surprisingly, such relocalization was not observed in adherens junction complexes (PECAM-1 and β-catenin). Taken together, these observations are consistent with observations in RBE4 monolayers [9] and suggest the ability of IMR90-derived BMECs to respond to hypoxic stimulus in similar fashion to rodent-based in vitro models.

### Hypoxia induces barrier disruption in both IMR90-derived BMECs and hCMEC/D3 cell monolayers

**Fig. 3:**
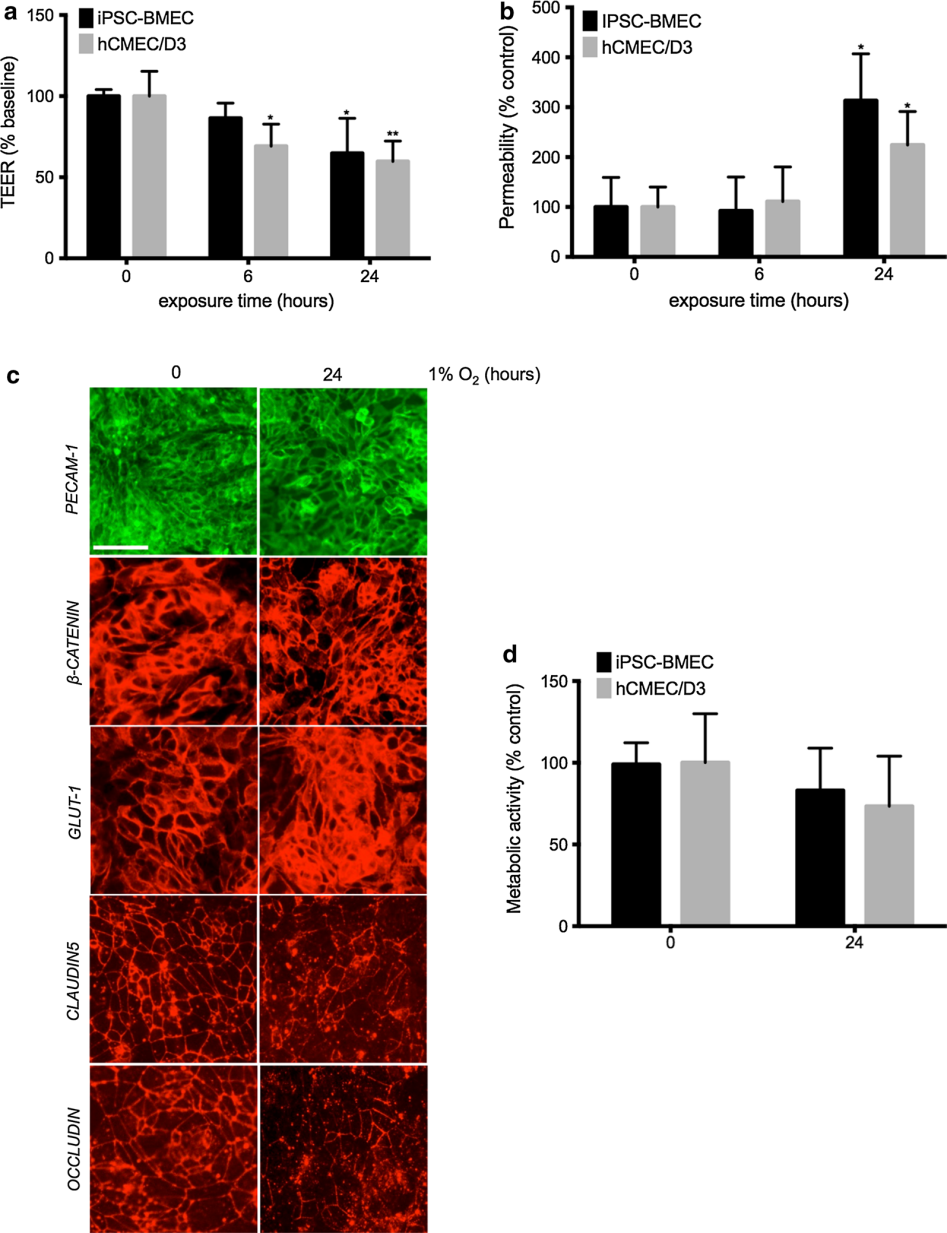
Prolonged Hypoxia impairs the barrier function in both cell monolayers. TEER (**a**) and sodium fluorescein (**b**) permeability values in both iPSC-BMECs and hCMEC/D3 cell monolayers following exposure to hypoxia for 6 and 24 h. Note the early onset of hypoxia-induced barrier disruption in hCMEC/D3 at 6 h as indicated by TEER compared to IMR90-derived BMECs, with a significant decrease at 24 h. Hypoxia-induced paracellular permeability however was only observed after 24 h in both groups. **c** Immunocytochemistry profile of IMR90-derived BMECs following exposure to hypoxia (1 % O_2_) for 24 h. Note the decreased immunoreactivity of claudin-5 and occludin following hypoxia, whereas no changes where noted in other cell junction proteins (β-catenin and PECAM-1). *Scale bar* = 20 µm. **d** Cell metabolic activity as measured by MTS assay. Following hypoxic incubation, MTS was added to the cell conditioned medium and incubated for 1 h before readout. Mean ± SD, n = 3 for each group. * and ** denotes *P* < 0.05 and *P* < 0.01 to controls (labeled as 0 h) respectively

Hypoxia-induced BBB disruption is a well-established phenomenon reported in both in vitro and in vivo models [9, 38–44]. Therefore, we assessed the ability of both IMR90-derived BMECs and hCMEC/D3 to respond to hypoxic stress by exposing our monolayers to 1 % O_2_ for 6 or 24 h (Fig. 3).

Acute (6 h) hypoxic stress resulted in significant decrease of over 70 % in TEER in hCMEC/D3 monolayers (Fig. 3a), whereas IMR90-derived BMECs showed only a mild decrease. However, such changes in TEER were not reflected in changes in vascular permeability, as we noted no significant increase in sodium fluorescein permeability (Fig. 3b).

In contrast, prolonged hypoxic (24 h) injury resulted in loss of barrier function in both IMR90-derived BMECs and hCMEC/D3, as we observed a 50 % decrease in TEER (Fig. 3a) and a 2.5-fold increase in fluorescein permeability (Fig. 3b) compared to normoxic monolayers. These results suggest the ability of the monolayers to respond to hypoxia similarly to the existing literature.

To further understand how hypoxia affects the barrier function, we investigated changes in cell junction complexes in IMR90-derived BMECs by immunocytochemistry (Fig. 3c). After 24 h of hypoxia, we did not observe any changes in adherens junction (PECAM-1 and β-catenin) complexes. However, we noted changes in tight junction (claudin-5 and occludin) complexes with a discontinuous staining pattern. Finally, we assessed by MTS whether this insult impacted cell viability (Fig. 3d). Following 24 h of hypoxic stress, we did not find any significant changes in cell metabolic activity, as the average metabolic activity was 80 and 70 % of the normoxic levels in IMR90-derived BMECs and hCMEC/D3 monolayers, respectively. In conclusion, our data demonstrate that IMR90-derived BMECs, as well as hCMEC/D3 cell monolayers, actively respond to hypoxia by disruption of their barrier function. Such disruption appears driven by alterations in the tight junction complexes rather than cell death.

### Oxygen-glucose deprivation impairs barrier function in both IMR90-derived BMECs and hCMEC/D3 monolayers

**Fig. 4:**
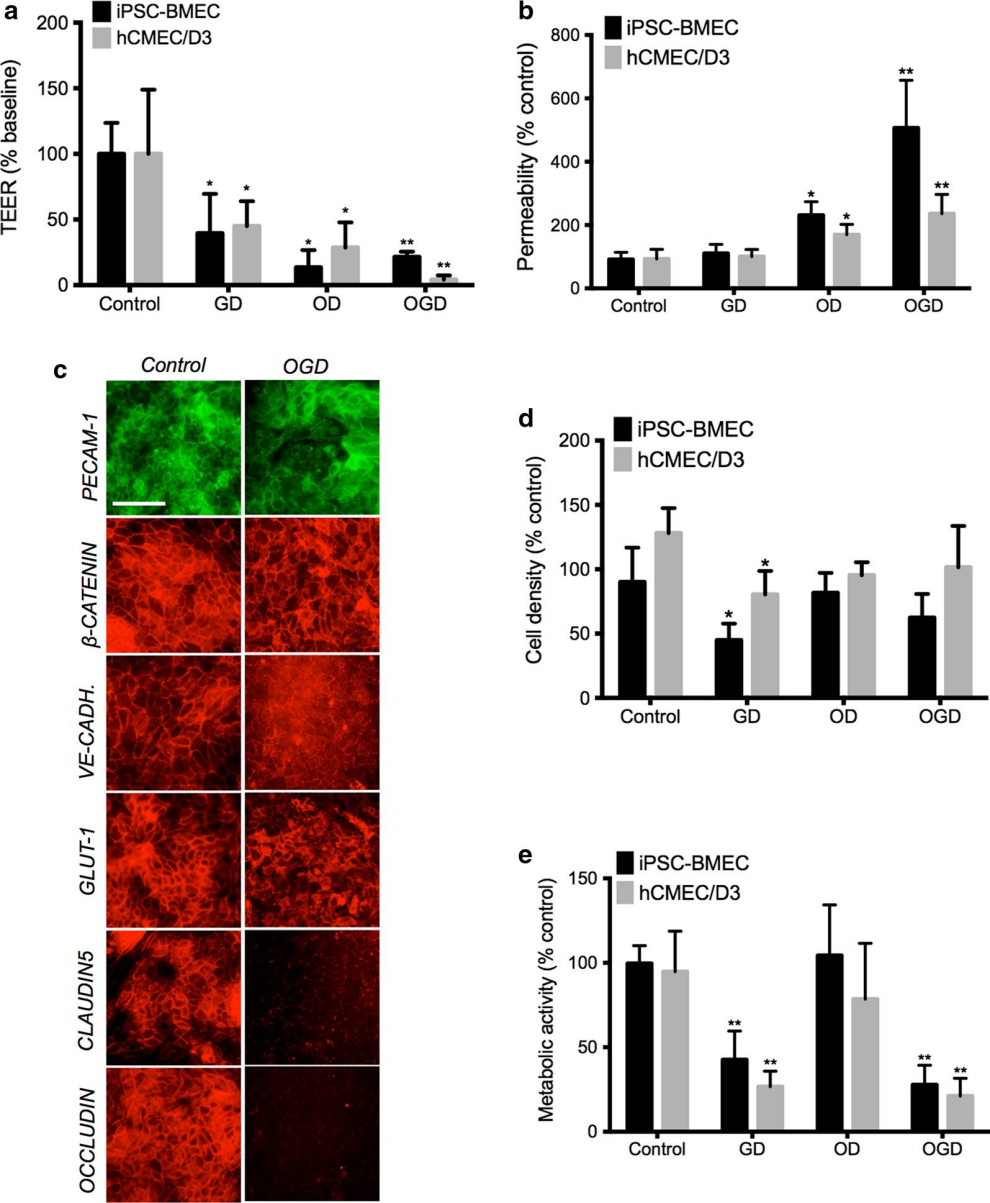
OGD induced barrier disruption in human BMECs.TEER (**a**) and sodium fluorescein (**b**) permeability values in both iPSC-BMECs and hCMEC/D3 cell monolayers following aglycemic (GD), hypoxic (OD) and oxygen-glucose deprivation (OGD) stress. Cells incubated in DMEM with glucose under normoxia served as control. Note the increase in fluorescein permeability following OD and OGD stress. **c** Representative immunocytochemistry pictures in iPSC-derived BMECs monolayers following 24 h of OGD stress. Note the overall degradation in cell junctions, with a quasi-disappearance in TJ complexes immunoreactivity. *Scale bar* = 20 µm. **d** IMR90-derived BMECs and hCMEC/D3 average cell density following treatment with aglycemia, hypoxia or OGD stress. Notably, aglycemia (GD) showed the lowest cell density in both cell monolayers. **e** Cell metabolic activity following 24 h of treatment. After 24 h of treatment, MTS reagent was added to conditioned cell medium and allowed to further incubate for 1 h. Mean ± SD, n = 3 for each group. * and ** denotes *P* < 0.05 and *P* < 0.01 to controls (DMEM with glucose, normoxia) respectively

Next, we investigated how hypoxia/aglycemia affected the barrier function in our human models of the BBB by exposing our cells to hypoxia, aglycemia or to oxygen-glucose deprivation (OGD) stress (Fig. 4). Interestingly, incubation in glucose-deprived medium (GD) for 24 h resulted in a significant decrease in TEER (~50 %) in both hCMEC/D3 and IMR90-derived BMEC monolayers (Fig. 4) but failed to show any significant increase in cell permeability to sodium fluorescein (Fig. 4b) suggesting that aglycemia alone was not sufficient to induce BBB disruption. On the other hand, hypoxia (OD) was capable of inducing BBB disruption, as we noted a significant decrease in TEER and an increase in when fluorescein permeability. However, we observed the maximum disruption cells were subjected to both oxygen and glucose deprivation (OGD), as noted by the lowest TEER values (20 % of control) and highest permeability values to fluorescein (5-fold increase).

Following such observations, we investigated changes in cell junction complexes in IMR90-derived BMECs following OGD stress by immunocytochemistry (Fig. 4c). OGD stress was capable of altering adherens junction complexes (as noted by a decrease in VE-cadherin immunoreactivity) but more importantly, we noted a complete loss of immunoreactivity for tight junction complex proteins.

Taken together, loss of tight junction complexes during OGD stress may be a contributor to the BBB disruption.

As changes in cell density and viability may impact the barrier integrity during injury, we investigated changes in cell density by trypan-blue exclusion assay (Fig. 4d). Interestingly, we noted a significant decrease in cell density after aglycemic treatment in both IMR90-derived BMECs and in hCMEC/D3 monolayers. However, we did not notice a significant decrease in cell density following hypoxia and OGD stress (two conditions shown to have an impact on the barrier function). Furthermore, we observed a similar outcome when we measured changes in cell viability by MTS assay (Fig. 4e). Aglycemia decreased metabolic activity level by 60 % compared to controls in both monolayers. However, hypoxia (OD) alone showed no differences compared to controls. Finally, cells exposed to OGD stress showed only 15–20 % of the metabolic activity observed in the control group. Taken together, our study demonstrates that both IMR90-derived BMECs and hCMEC/D3 respond to OGD stress with disrupted barrier function. However, we noted an uncoupling between BBB barrier function and cell density/cell metabolic activity following hypoxic or aglycemic treatment.

## Discussion

Stroke constitutes the fifth leading cause of death and it is a major cause of disability in the United States. Although important efforts have been made to identify therapeutics capable of improving outcome in stroke patients, the ability to translate findings from animal models to patients has had little success.

Human Induced pluripotent stem cells (iPSCs) based in vitro models of the neurovascular unit (integrating BMECs, astrocytes and neurons) may help provide a screening platform to improve such translation. However, pluripotent stem cells are also highly hypoxic tolerant [45], therefore questioning the suitability of stem-cell based models for understanding the effects of hypoxic/ischemic injury in vitro.

In this study, we investigated the ability of stem cell derived BMECs to respond to hypoxic/ischemic injury in vitro by comparing their response to hCMEC/D3, an immortalized adult human BMEC line [22]. We first investigated changes in the barrier function following CoCl_2_ treatment, a chemical commonly used to simulate a hypoxic response in mammalian cells [25, 26, 28, 29]. We noted a significant decrease in barrier function in IMR90-derived BMECs and a loss of monolayer integrity. Such results are in agreement with a previous study by Engelhardt and colleagues [9]. In that study, the authors observed that CoCl_2_ was capable of significantly decreasing barrier function in RBE4 cells monolayers as early as 6 h. In contrast to their findings, we observed a significant decrease in the barrier function only after 24 h in IMR90-derived BMECs. Surprisingly, we did not observe a response in hCMEC/D3 monolayers following CoCl_2_ exposure, with no changes in barrier function compared to untreated groups.

A difference in response to CoCl_2_ between the two monolayers might be explained by differences in oxygen sensing, as CoCl_2_ exerts its activity through the activation of the hypoxia-induced factor (HIF)-1 pathway. Such differences maybe also inherent to the nature of the hCMEC/D3 cells, as Patak and colleagues reported an absence of up-regulation in ABCB1 and ABCC1 expression following hypoxic stress [46]. We are currently investigating such dimorphism in oxygen sensing between these two cells by comparing HIF-1α expression at protein levels and VEGF production.

Following these observations, we investigated the cellular response to environmental hypoxia by incubating cells in the presence of 1 % O_2_, a gas phase concentration commonly used in the literature. In our study, both IMR90-derived BMECs and hCMEC/D3 cells responded to prolonged hypoxic injury in vitro with decreased barrier function. Furthermore, IMR90-derived BMECs displayed alterations in tight junction complex distribution similar to previous reports [9]. However, oxygen-glucose deprivation (OGD) stress was necessary to achieve a major barrier disruption in both IMR90-derived BMECs and hCMEC/D3 cells. Such disruption was marked by the greatest decrease in TEER and highest increase in fluorescein permeability.

This dramatic decrease in barrier function was likely due to a major disruption in tight junction complex integrity, as we noted a significant decrease in immunoreactivity of both claudin-5 and occludin in IMR90-derived BMECs. We speculate such decreased immunoreactivity may be indicative of a cleavage of such proteins by a select number of matrix metalloproteinases (MMPs), in particular by MMP-2 and MMP-9 [47–49].

In addition to changes in barrier function due to changes in MMP activity, we also speculate that impaired energy production during hypoxic/ischemic injury may contribute to barrier disruption. A recent study by Vandekeere and colleagues suggests ATP production in brain endothelial cell metabolism is driven by glycolysis under aerobic conditions [50]. MTS-based assays are designed to measure changes in mitochondrial hydrogenase activity [51]. Thus, we were expecting little change in MTS activity following hypoxic/OGD stress in our model. Surprisingly, we noted no changes in MTS activity in hypoxic cells. Such results were consistent with a recent study published by Ogunshola and colleagues [10], in which no significant changes in cell metabolism were observed following prolonged hypoxia.

Interestingly, we noted changes in cell density following CoCl_2_ treatment as well as during glucose deprivation. Although a decrease in cell density was accompanied by an increase in permeability following CoCl_2_ treatment, we did not observe such phenomenon in glucose-deprived cells.

Finally, we observed a conundrum between metabolic activity and barrier function in BMECs, in particular between hypoxic and aglycemic treatments. Decreased cell metabolism in aglycemic cells was not accompanied by decreased barrier function, whereas we noted a loss of barrier function was not accompanied by a decrease in cell metabolism. Such data suggests that tight junction disruption following hypoxia may not be due to energy deficiency, but also suggests that IMR90-derived BMECs may utilize other nutrients to maintain mitochondrial activity through some anaploretic reactions (in particular glutamine). Thus, we are currently investigating how hypoxia/OGD stress influences energy production in BMECs; in particular we are investigating how such stress influences changes in glycolysis and oxidative phosphorylation mechanism.

## Conclusions

In summary, this study demonstrates the suitability of stem cell-derived in vitro model of the human BBB as an in vitro model for H/I. This model may effectively complement existing in vivo models and help improve the identification of novel therapeutics capable of fighting H/I-induced BBB disruption following stroke.

## Additional file

10.1186/s12987-016-0042-1 Representative diagram of the iPSC-derived BMEC differentiation protocol.

## Abbreviations

BBB: blood-brain barrier
BMECs: brain microvascular endothelial cells
DMEM: Dulbecco’s modified eagle medium
GD: glucose deprivation
H/I: hypoxia-ischemia
OD: oxygen deprivation
OGD: oxygen-glucose deprivation
PBS: phosphate-buffered saline solution
TEER: transendothelial electrical resistance
TJ: tight junctions
