# Supplementary figures and images for: Cerebral hypoxia/ischemia selectively disrupts tight junctions complexes in stem cell-derived human brain microvascular endothelial cells
Source: Fluids Barriers CNS. 2016 Oct 11;13:16. doi: 10.1186/s12987-016-0042-1 (PMC5057206; doi:10.1186/s12987-016-0042-1)

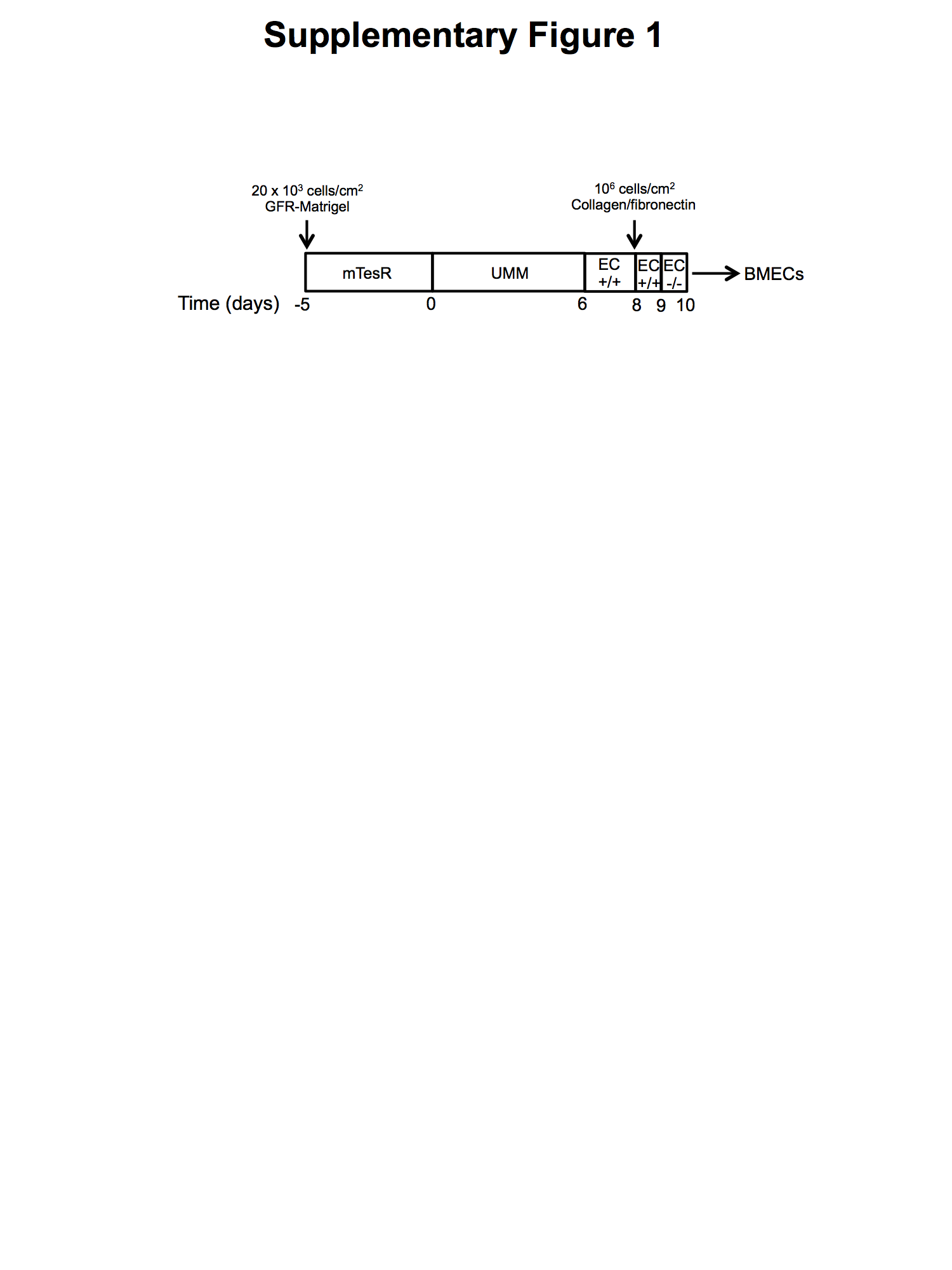

Supplement: Supplementary file 1 — 10.1186/s12987-016-0042-1 Representative diagram of the iPSC-derived BMEC differentiation protocol. [file 12987_2016_42_MOESM1_ESM.tiff]
